# The Influence of Transcranial Alternating Current Stimulation on the Excitability of the Unstimulated Contralateral Primary Motor Cortex

**DOI:** 10.3390/brainsci15050512

**Published:** 2025-05-17

**Authors:** Erik W. Wilkins, Richard J. Young, Ryder Davidson, Reese Krider, George Alhwayek, Jonathan A. Park, Armaan C. Parikh, Zachary A. Riley, Brach Poston

**Affiliations:** 1 Department of Kinesiology and Nutrition Sciences, University of Nevada-Las Vegas, Las Vegas, NV 89154, USA; wilkie1@unlv.nevada.edu (E.W.W.); parkj62unlv@gmail.com (J.A.P.); 2 Interdisciplinary Ph.D. Program in Neuroscience, University of Nevada-Las Vegas, Las Vegas, NV 89154, USA; ryoung@unlv.edu; 3 School of Medicine, University of Nevada-Las Vegas, Las Vegas, NV 89154, USA; davidr6@unlv.nevada.edu (R.D.); krider1@unlv.nevada.edu (R.K.); alhwag1@unlv.nevada.edu (G.A.); 4 Department of Special Education, Vanderbilt University, Nashville, TN 37212, USA; armaan.c.parikh@vanderbilt.edu; 5 Department of Kinesiology, Indiana University Purdue, Indianapolis, IN 46202, USA; zariley@iupui.edu

**Keywords:** transcranial alternating current stimulation, transcranial direct current stimulation, transcranial magnetic stimulation, cortical excitability, motor evoked potential, motor skill

## Abstract

Objectives: Transcranial alternating current stimulation (tACS) can enhance primary motor cortex (M1) excitability and improve motor skill when delivered unilaterally to the dominant hemisphere. However, the impact of tACS on contralateral M1 excitability both during and after application has not been studied. The purpose of this study was to examine the effects of tACS delivered to the dominant left M1 on the excitability of the unstimulated contralateral non-dominant right M1. Methods: This study implemented a double-blind, randomized, SHAM-controlled, within-subjects, crossover experimental design. Eighteen young adults completed a tACS condition and a SHAM condition on two different days in counterbalanced order with a week washout period between days. Transcranial magnetic stimulation (TMS) was utilized to assess excitability of the contralateral right M1 while tACS was delivered to the left M1. TMS was administered in five test blocks (termed Pre, D5, D10, D15, and Post) relative to a 20 min application of tACS (70 Hz, 1 mA current strength). The Pre and Post TMS test blocks were conducted before and immediately after tACS was applied to the left M1, whereas the TMS test blocks performed during tACS were completed at time points starting at the 5, 10, and 15 min marks of the 20 min stimulation period. The primary dependent variable was the 1 mV motor evoked potential (MEP) amplitude. MEP data were analyzed with a 2 *condition* (tACS, SHAM) × 5 *test* (Pre, D5, D10, D15, Post) within-subjects ANOVA. Results: The main effect for *condition* (*p* = 0.704) and *condition* × *test* interaction (*p* = 0.349) were both non-statistically significant. There was a significant main effect for *test* (*p* = 0.003); however, post hoc analysis indicated that none of the pairwise comparisons were statistically significant. Conclusions: Overall, the findings indicate that tACS applied to the left M1 does not significantly modulate contralateral right M1 excitability during or immediately after stimulation, at least when utilizing the present tACS parameters.

## 1. Introduction

Transcranial alternating current stimulation (tACS) is a non-invasive brain stimulation method that has been receiving increasing research attention over the past several years [1,2,3,4,5,6,7]. tACS is a variation of the much more commonly studied non-invasive brain stimulation technique of transcranial direct current stimulation (tDCS). tDCS involves passing a weak, constant current between a cathode and an anode placed on the scalp [8,9]. In contrast, tACS involves the application of a sinusoidal waveform at a given frequency via an anode and a cathode placed over two scalp areas to impact a target brain region or two functionally connected regions. Accordingly, tACS can be used to induce entrainment of populations of cortical neurons at the same frequency of oscillation as endogenous oscillations both within and between brain areas [1,10,11].

Despite these two basic differences between tDCS and tACS, the two techniques have some common features, methodological similarities, and can elicit a few basic overall net effects on physiological and behavioral outcomes relative to human motor performance. For example, two of the basic goals of the application of both techniques are to increase the excitability of the primary motor cortex (M1) and to induce improvements in motor skill acquisition [12,13,14,15]. This is most frequently accomplished in tDCS studies by placing the cathode over the right supraorbit (SO) and the anode over the contralateral left M1, which is termed the SO-M1 electrode montage. Most typically, a current of 1–2 mA in intensity is passed between the electrodes for a duration of 10–20 min. These tDCS parameters have shown the ability to increase M1 excitability by approximately 20–40% after stimulation [13,14,16,17] and induce improvements in motor skill of about 10–15% [8,9,12,13,14,15,18,19,20,21]. In contrast, fewer studies have investigated the influence of tACS on these outcomes and the parameters of stimulation have arguably been more heterogenous in tACS compared to tDCS studies. Nonetheless, several studies have reported that tACS can also increase M1 excitability [1,22,23], although perhaps not to the same degree as tDCS. In addition, a few studies have also demonstrated that tACS applied to M1 can significantly enhance fine motor skill in hand muscles. For instance, Sugata (2018) [2] found that 70 Hz tACS delivered to the left M1 using the SO-M1 montage and a 1 mA current elicited large improvements in motor skill in a four-finger button pressing task involving the right hand. The magnitude of improvements in that study seemed to indicate that the set of parameters employed may be among the most effective in the literature to date. Overall, the vast majority of M1-tDCS studies along with a few promising M1-tACS studies have shown a clear ability to significantly enhance cortical excitability and motor skill when using these sets of parameters and the SO-M1 electrode montage to target the dominant left M1 that predominately controls the contralateral dominant right hand [12].

In contrast to this large body of research, there is a paucity of studies that have explored the effects of M1-tDCS or M1-tACS on the excitability of unstimulated right non-dominant M1 and the motor performance of the associated left non-dominant hand that it predominately controls. For example, a previous study by another research group directly measured the influence of left M1-tDCS on the net excitability of the right M1 as measured by motor evoked potential (MEP) amplitudes elicited by transcranial magnetic stimulation (TMS) [24]. This study found that right M1 excitability was unchanged relative to baseline immediately and 40 min after left M1-tDCS application. Similarly, a recent study from our laboratory reported that tDCS applied to the left M1 did not significantly modulate right M1 excitability during or immediately after stimulation had ended [25]. Most importantly, however, no analogous studies exist that have investigated the effects of M1-tACS on right M1 excitability during or after stimulation in similar experimental circumstances. Similarly, only a few tDCS studies have investigated the motor performance related outcomes in response to application of left M1-tDCS using the SO-M1 montage on the ipsilateral left hand primarily controlled by the right M1. The outcomes were contradictory as some reported no effects on motor skill of the left hand [26,27], whereas another other found a trend for a decline in motor skill [28]. In addition, it appears that no analogous tACS studies have been conducted on these interrelated topics. Therefore, the impact of left M1-tACS on right M1 excitability and motor skill of the left hand remain to be elucidated.

The reasons for the scarcity of available research on these topics are difficult to determine given that left M1-tDCS given during task practice has generally been shown to yield superior motor skill outcomes in motor tasks executed with the right hand relative to before practice application [29,30,31]. Accordingly, it would seem that it would be important to know the effects of the stimulation on the right M1 during that time as it also contributes to the motor skill learning outcomes of the right during and after practice. This is because although the right hand is predominantly controlled by the contralateral left M1, the ipsilateral M1 plays a significant role in the control of movements of the ipsilateral hand that it does not predominantly control [32,33]. In fact, a large body of evidence provides support for the meaningful contribution of ipsilateral right M1 to important facets of motor control such as the initiation of movements [34,35,36] and ipsilateral right hand motor skill acquisition [37,38,39,40,41]. Therefore, it would also be valuable to characterize the influence of left M1-tACS delivered via the most common M1-SO montage on the unstimulated non-dominant contralateral right M1. Theoretically, modulations of right M1 under these conditions could have significant implications for movement control and motor skill learning related processes in both hands. However, a rational initial exploration of possible modulations of right M1 excitability during and after application of left M1-tACS would need to keep the target muscle at rest to isolate the fundamental effects of stimulation without the influence of concurrent muscle activation or performance of a motor task.

The purpose of this study was to examine the effects of tACS delivered to the dominant left M1 on the excitability of the unstimulated contralateral non-dominant right M1. This was achieved by quantification of MEP amplitudes obtained from the left first dorsal interosseous (FDI) muscle elicited by TMS delivered to the right M1 in test blocks conducted before, during, and after 70 Hz tACS was administered to the left M1 for a duration of 20 min. Based on the most relevant previous M1-tDCS and M1-tACS studies [1,22,42], it was predicted that MEP amplitudes elicited from the right M1 would be greater during and immediately after application of tACS to the left M1 relative to baseline and compared with the SHAM condition. The presence of modulations of unstimulated contralateral right M1 excitability by left M1-tACS could have implications for influencing the control of voluntary movements and motor learning processes in both the left and right hands.

## 2. Materials and Methods

### 2.1. Participants

Eighteen healthy young adults consisting of 10 males and 8 females with an average age of 25.9 ± 5.1 SD years participated in this study. Prior to their involvement, all volunteers provided written informed consent. Participants were strongly right-handed as indicated by the Edinburgh Handedness Inventory [43]. They were screened to ensure the absence of neurological or psychiatric disorders, uncontrolled medical conditions, and compliance with international criteria for non-invasive brain stimulation [44]. This study complied with the Declaration of Helsinki and received approval from the Biomedical Institutional Review Board at the University of Nevada, Las Vegas, NV, USA.

### 2.2. Experimental Design

The study implemented a double-blind, SHAM-controlled randomized, within-subjects, experimental design and had a nearly identical design to a previous study [25] from our laboratory that utilized tDCS. A within-subjects design was selected to preclude the influence of interindividual variations in physiological, genetic, and anatomical factors on the susceptibility to tDCS [45,46] while also allowing for greater statistical power compared to between-subjects designs [47]. The presentation order of the two experimental conditions was given to the participants using Research Randomizer (www.randomizer.org) by an investigator not involved in the data collection aspects of the experiments [48,49]. Each participant completed two experiments at approximately the same time of day and held a week apart [50,51], which is the typical washout period utilized in the majority of tDCS and tACS studies. Most importantly, the only difference between the two experiments was the type of stimulation (tACS, SHAM) delivered.

**Figure 1:**
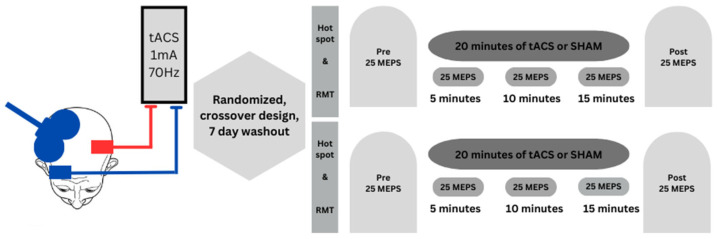
Schematic of the study design and experimental protocol. TMS coil positioning over the right M1 scalp area corresponding to the left FDI motor hotspot and the SO-M1 electrode montage placement for application of tACS over the left M1. A total of 20 min of tACS or SHAM stimulation was given to the left M1 using the M1-SO electrode montage. MEPs were elicited from the right M1 and acquired from the corresponding left FDI in five TMS test blocks (Pre, D5, D10, D15, Post) conducted before (Pre), during (D5, D10, and D15 min points in time), and immediately after (Post) either tACS or SHAM stimulation.

Each experimental session had a duration of ~1.5 h and the procedures were performed in the order prescribed: (1) the motor hotspot site, resting motor threshold (RMT), 1 mV MEP stimulation intensity (SI) expressed as a percentage of maximum stimulator output (% MSO) for the right FDI were all determined via TMS applied to the left M1; (2) placement of the tACS electrode montage over the left M1 FDI motor hotspot site; (3) the motor hotspot site, resting motor threshold (RMT), 1 mV MEP stimulation intensity (SI) expressed as a percentage of maximum stimulator output (% MSO) for the left FDI were all determined via TMS applied to the right M1; (4) Pre TMS test block; (5) tACS or SHAM stimulation was delivered to the left M1 for 20 min while three TMS test block were completed during (termed D5, D10, and D15) stimulation. Accordingly, TMS administration began at the 5, 10, 15 min points in time and lasted for ~2.5 min; and (6) Post TMS test block. MEPs collected during the TMS test blocks were always elicited from the right M1 and acquired from the corresponding left FDI. This experimental protocol is depicted in Figure 1.

### 2.3. Experimental Procedures

#### 2.3.1. TMS Administration and Surface EMG Recording

A monophasic Magstim 200^2^ (Magstim Company Ltd. Carmarthenshire, UK)unit with a standard double 70 mm figure-of-eight remote-controlled coil was employed to deliver single TMS pulses to the left and right M1s. The TMS coil was placed over the M1 of interest over the surface of the scalp with the handle directed backward and lateral while being orientated tangential to the scalp at a 45-degree angle relative to the sagittal plane. This coil positioning relative to the M1 hand representation area generates a posterior-to-anterior current direction in the brain to elicit MEPs in the contralateral hand. Surface electromyographic (EMG) activity of the FDI muscle of each hand was collected utilizing two electrodes configured in a belly tendon montage. The ground electrode comprised a thin circular disc and was placed on the back of the hand. EMG activity was collected with Cambridge Electronic Design (Cambridge, UK) hardware (1902 amplifiers, micro 1401 data acquisition interface) and software (Signal 5.04).

TMS administration and EMG signals were collected while participants a constant body and upper limb posture. The experimental arrangement was the same as previous studies involving TMS of the right FDI [25,31,52] and MEPs acquired from the left FDI was accomplished in an analogous manner. In both cases, participants sat in a chair with the upper arm abducted to about 45-degrees with the forearm and elbow placed on the surface of a table located to the side of the body. The wrist was maintained in a neutral position, the elbow was flexed to about a 90-degree angle, and the palm was placed flat on the table surface. The participants were given strict instructions to keep this posture for all TMS testing procedures as even relatively modest differences in arm position can significantly influence the MEP amplitudes observed in hand muscles [53,54]. Crucially, participants were directed to keep their eyes open during TMS as closure of the eyes is well-known to significantly diminish MEP amplitude compared to an eyes open state [55]. To make sure that the FDI muscle was kept in a state of complete rest during all TMS testing, real-time visual feedback [56] of FDI EMG was given on a large monitor situated about ~0.75 m directly in front of the participants [57]. A set of instructions was given to participants on how to utilize the visual feedback provided to keep the FDI in a state of rest throughout the experiment. One member of the research team had the sole task of constantly monitored the body positioning and FDI EMG activity of participants to further assure that these requirements were met throughout the experiments.

The motor hotspot site, RMT, and 1 mV MEP stimulation intensity (SI) as a % MSO were determined for the right FDI followed by the left FDI [25]. The motor hotspot sites were located by delivering a series of TMS pulses over the scalp of the hand representation area of each M1. This was continued until the point that elicited the greatest MEP amplitude in the corresponding FDI was identified [58]. This point was used as the stimulation site and marked with a temporary pen for all of the following RMT and 1 mV SI measurements as well as the assessment of MEP amplitudes in the TMS test blocks. More specifically, the point was marked directly on the scalp for left M1 TMS testing, whereas it was marked on tape that was placed on the scalp cap for right M1 testing. Moreover, the outline of the coil was marked on the scalp cap and a line was placed on the forehead of the participants right beneath the edge of the scalp cap. This allowed for the coil position to be maintained relative to the FDI hotspot. RMT was quantified as the lowest TMS SI that could generate a MEP amplitude above 50 microvolts in at least 5 out of 10 successive trials. RMT data was collected for two reasons. First, a few studies [59,60] have reported that participants with a higher RMT may exhibit a smaller enhancement of MEPs after tDCS is applied and vice versa. This could also be the case for tACS, which may mean that RMT values could correlate with the percentage increase in MEP amplitude, if it were to occur. Second, RMT also served as a basic control measure and indication of baseline excitability for each FDI in both experiments. For the determination of the 1 mV MEP SI for the left and right M1s, a series of procedures developed in previous studies was used [25,31,52] as they proved very successful in being able to obtain the TMS SI (% MSO) that induced an average MEP of as close to 1 mV as possible for a block TMS trials. This is crucial because a significant difference in the MEP acquired at baseline (Pre TMS block) between the two experimental conditions could represent a potential confound and ideally would be as close to 1 mV as possible. In brief, this involved applying a series of TMS pulses starting at ~55% MSO and adjusting the SI while MEPs were simultaneously displayed and quantified online by the data acquisition software. Once MEP amplitudes appeared to be as close to an average value of 1 mV as possible, the data acquisition program was reset and data collection of the Pre TMS test block commenced. This ensured that the MEP amplitude attained in the Pre TMS test block was as close to 1 mV as practically possible in both stimulation conditions performed on the two separate days. The same 1 mV SI as a % MSO was utilized to elicit MEPs in the subsequent D5, D10, D15, and Post TMS testing blocks for each participant on a given day. Lastly, the inter-trial interval (ITI) between successive MEPs was 6 s for all TMS test blocks and 25 MEPs were collected per block as this has been shown to be the optimal trade-off between time efficiency and the ability to obtain valid MEP amplitude averages in blocks of TMS trials [61].

#### 2.3.2. tACS Administration and Electrode Placement

A NeuroConn DC Stimulator Plus/MR (Neurocare Group AG, Ilmenau, Germany) was utilized to deliver high frequency (70 Hz) tACS for a duration of 20 min with a current strength of 1 mA to the left M1. This was conducted using the traditional M1-SO electrode montage that has been used in the majority of tDCS studies and many tACS studies. The two rubber electrodes (5 × 7 cm^2^) that comprised the electrode montage were inserted into two sponged that were in sponges doused in saline solution. The reference electrode was placed above the eyebrow over the right supraorbital region and the target electrode was placed over right FDI motor hotspot site of the left M1. To accomplish the main experimental goal of collecting MEPs from right M1 while tACS was simultaneously applied to the left M1, a set of novel procedures were implemented after extensive pilot work. The target and reference electrodes were fixed in place by a close-fitting scalp cap as opposed to usual employment of two separate rubber straps consisting of a headband and a chinstrap. Specifically, the target electrode was kept in place by the scalp cap only and the typical rubber chin strap was not used for this electrode. This was necessary as this would have clearly interfered with the concurrent TMS measurements as the strap would have traversed directly over the right M1 motor hotspot site. Overall, the close-fitting scalp cap was able to successfully keep the target electrode in place to the same extent as the conventional rubber strap positioning. In contrast, the cathode was able to further secured using the typically applied rubber strap placed around the head in the conventional manner as this rubber strap was not in a position to interfere with the TMS testing. These methods for securing the tACS electrodes was ultimately effective because while the TMS coil was in relatively close proximity to the electrodes, it did not contact them. For the SHAM stimulation condition, the standard [31,62] ramp up, hold, ramp down protocol was performed. Accordingly, the current was ramped up from zero to 1 mA over 10 s, maintained at 1 mA for 30 s, and ramped down from 1 mA to zero over 10 s as in previous studies. This is the most common SHAM stimulation paradigm used in tDCS and tACS studies and most studies have found that it induces the same sensations on the scalp to participants as real stimulation, but does not induce any physiological effects [19,63]. Finally, the research team member responsible for operated the tACS device did not partake in the data collection aspect of the experiments and research team members that conducted the data collection were blind to the stimulation condition as in previous studies [31,48,49].

### 2.4. Data and Statistical Analyses

The dependent variables were MEP amplitude, RMT, and 1 mV SI. MEP amplitude was the primary dependent variable, whereas the secondary dependent variables of RMT and 1 mV SI were considered experimental control measures. Signal software (version 5.04) from Cambridge Electronic Design (Cambridge, UK) was used to collect all EMG signals and associated MEP data via custom-written scripts. In addition, offline data analysis of MEP data was also accomplished using custom-written Signal scripts. MEP amplitude was calculated as the peak-to-peak values and the average of the 25 MEPs [61] collected in each of the TMS test blocks was taken for analysis.

Two separate 2 *condition* (tACS, SHAM) × 2 *hand* (Left, Right) within-subjects ANOVAs were used to analyze the control measures of RMT and 1 mV SI. The primary dependent variable of MEP amplitude was analyzed with a 2 *condition* (tACS, SHAM) × 5 *test* (Pre, D5, D10, D15, Post) within-subjects ANOVA. If appropriate, post hoc analyses were conducted utilizing Bonferroni adjustments for multiple comparisons to determine where any statistically significant differences occurred between the TMS test blocks. The effect sizes were reported as the partial eta squared values. The level of significance for the statistical tests was *p* < 0.05. Data were depicted as means ± standard errors in the figures, whereas data referred to in the text were expressed as means ± standard deviations.

## 3. Results

### 3.1. Control Variables: RMT and 1 mV SI

**Figure 2:**
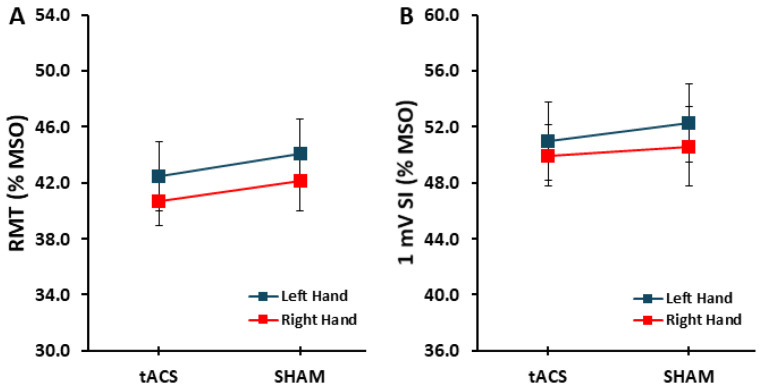
RMT and 1 mV SI. (**A**) RMT in the left and right hands for the tACS and SHAM conditions; (**B**) 1 mV SI in the left and right hands for the tDCS and SHAM conditions.

The *condition* main effect (*F*_1,17_ = 1.612, *p* = 0.221, η_p_^2^ = 0.087), *hand* main effect (*F*_1,17_ = 2.848, *p* = 0.110, η_p_^2^ = 0.114), and *condition* × *hand* interaction (*F*_1,17_ = 0.033, *p* = 0.857, η_p_^2^ = 0.002) were all not statistically significant (Figure 2A). For the 1 mV SI, the *condition* main effect (*F*_1,17_ = 0.718, *p* = 0.409, η_p_^2^ = 0.041), *hand* main effect (*F*_1,17_ = 0.053, *p* = 0.821, η_p_^2^ = 0.003), and *condition* × *hand* interaction (*F*_1,17_ = 0.153, *p* = 0.700, η_p_^2^ = 0.009) were all not statistically significant (Figure 2B).

### 3.2. MEP Amplitude

**Figure 3:**
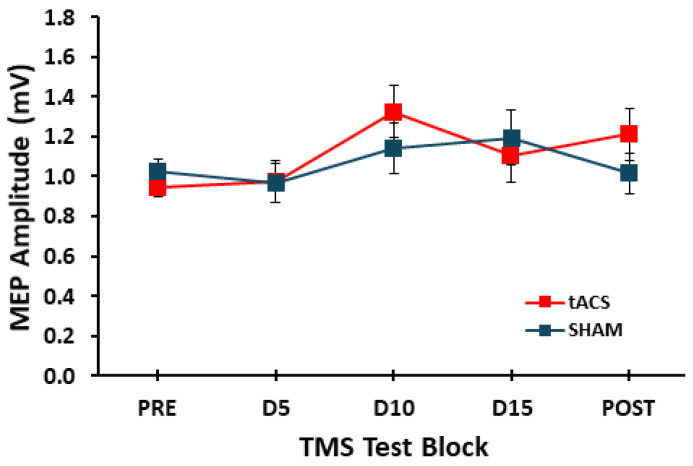
MEP amplitudes evoked from the right M1 and acquired from the left hand in the tACS and SHAM conditions for the Pre, D5, D10, D15, and Post TMS test blocks.

For MEP amplitude, the *condition* main effect (*F*_1,17_ = 0.149, *p* = 0.704, η_p_^2^ = 0.009) and *condition* × *test* interaction (*F*_4,68_ = 1.111, *p* = 0.349, η_p_^2^ = 0.061) were both not statistically significant. In contrast, there was a significant main effect for *test* (*F*_4,68_ = 4.424, *p* = 0.003, η_p_^2^ = 0.207; Figure 3). However, post hoc analyses of the main effect indicated that none of the pairwise comparisons were statistically significant (*p* value range 0.066–1.000).

## 4. Discussion

The purpose was to examine the effects of tACS delivered to the dominant left M1 on the excitability of the unstimulated contralateral non-dominant right M1. The primary findings were that MEP amplitudes elicited from the right M1 and acquired from the left hand were not significantly different between the tACS and SHAM conditions at any of the points in time during or immediately after the stimulation was given to the left M1. Overall, the findings indicate that tACS applied to the left M1 does not significantly modulate contralateral right M1 excitability during or immediately after stimulation, at least when the current set of tACS parameters are used.

### 4.1. Effects of tACS on Excitability of the Contralateral Non-Dominant M1

tACS delivered to the left M1 using the SO-M1 electrode montage usually enhances left dominant M1 excitability, as assessed by MEPs acquired from the contralateral right hand that it predominantly controls [1,22,23]. To the best of our knowledge, this was the first study to investigate the influence of left M1-tACS on the excitability of the unstimulated right non-dominant M1. It was based on a previous study from our laboratory using a nearly identical design except that tDCS was used [25]. In particular, the most innovative feature of the current study was the quantification of right M1 excitability simultaneous with left M1-tACS administration. Accordingly, our previous study that used this novel methodology is the only M1-tDCS study available that directly measured the influence of left M1-tDCS on the excitability of the right M1 [25] simultaneous with the stimulation. However, a previous study by Lang and colleagues [24] evaluated this issue before and after tDCS but not during tDCS application. Most importantly, no studies have performed analogous assessments of right M1 excitability during or after M1-tACS of the left M1. As a result, all but one study [25] involving either technique have measured net excitability of the stimulated left M1 before and after the two types of stimulation were applied. This makes sense based on basic technical constraints related to the fact that MEPs cannot be evoked from the stimulated M1 while that target tACS electrode is over that scalp location during stimulation. However, the current study used a novel arrangement involving the use of a scalp cap and the removal of the typical chinstrap that transverses the head over the right M1 motor hotspot to successfully apply TMS to the right M1 during tACS application to the left M1 as was performed with tDCS in our prior related study [25].

The initial hypothesis proposed that MEP amplitudes elicited from the right M1 would be higher during and immediately after application of tACS to the left M1 relative to baseline and compared with the SHAM condition. Since no prior tACS studies had investigated these exact topics, this hypothesis was primarily based on the most applicable two tDCS studies available that had explored contralateral right hemisphere responses to left M1-tDCS [24,42]. One study used MRS to quantify levels of GABA in the non-stimulated right M1 and the stimulated left M1 [42]. The results revealed that GABA levels declined in both M1s which would imply that both M1 displayed less inhibition and therefore higher excitability following left M1-tDCS. However, concurrent TMS measures of MEP amplitude were not included in that study to confirm that the net excitability of each both had in fact increased. This could be necessary as other neuromodulator concentrations could have changed due to tDCS and counteracted the inhibitory effects of increased GABA concentrations [9,64]. In contrast to the original hypothesis, MEPs evoked from the right M1 and acquired from the associated left hand were not significantly different between the tACS and SHAM conditions during or immediately following left M1 stimulation. Furthermore, none of the TMS test blocks performed during or immediately after stimulation exhibited MEP amplitudes that significantly differed relative to the Pre TMS test block, independent of stimulation condition, for either of the two stimulation conditions. Therefore, the overall results were similar to our previous tDCS study that also found no modulations in right M1 excitability either during or after tDCS applied to the left M1 [25].

However, the statistical results that led to these overall conclusions in the present study were more nuanced than portrayed above. Specifically, while there was no main effect for *condition* or *condition* × *test* interaction. The ANOVA did reveal a significant *test* main effect. However, post hoc analyses using Bonferroni corrections indicated that none of the pairwise comparisons were statistically significant, although the differences between the D10 TMS test block compared to the D5 and the Pre TMS test blocks had *p* values of 0.066 and 0.082, respectively. In addition, visual inspection of Figure 3 and the average MEP values in the tACS and SHAM conditions, indicate that the non-significantly higher MEP amplitudes in the D10 TMS test block were due to a combination of higher MEP amplitudes in both stimulation conditions. Therefore, this pattern of results complicates the interpretation of the results to a certain extent. Since MEP amplitudes were moderately increased in D10 for the tACS, it would be tempting to conclude that left M1-tACS may have had an effect on right M1 excitability that was slightly masked by the unexpected, but non-significant increased MEP values in the D10 test block in the SHAM condition. Despite these statistically nuanced results, we feel that the overall interpretation of no significant *test* effect due to non-significant post hocs and no significant tACS effects are the correct overall conclusions. Accordingly, the rather high D10 TMS test block MEP values were most likely due to random fluctuations in MEP measurements due to their well-described and notorious inherent variability [61,63], and not any actual effects due to tACS or SHAM stimulation. Nonetheless, it cannot be completely ruled out that placebo effects could have provided at least some contribution to the enhanced MEPs in both stimulation conditions, but especially the tACS condition [63]. Specifically, as discussed in a review by Dissanayaka and colleagues [63], tACS may be more likely to induce placebo effects compared with tDCS. This is because tACS can produce phosphenes and sensations of flickering in almost one half of participants, although most of the studies on this topic were performed with different tACS parameters compared to the current study. Finally, the overall findings could not have been influenced by any potential confounding effects as the control measurements of RMT and the 1 mV SI did not differ significantly between the tACS and SHAM conditions. Critically, the 1 mV SI (% MSO) yielded average MEPs very close to the ~1 mV target for the pre TMS test blocks, which eliminated the greatest potential confound of the study.

Our results also potentially conflict with predictions from modeling studies of current flow and the electric field generated using the SO-M1 electrode montage in both tDCS and tACS studies. For example, a review article [65] showed (their Figure 1A) that tACS of the left M1 creates an electric field affecting the right M1 and several connected contralateral and ipsilateral brain regions. One would expect this to modulate right M1 excitability, either increasing or decreasing it. The absence of any modulation in MEP amplitudes in this study suggests that the current flow pattern did not result in any net change in the balance of inhibitory and excitatory circuits in the right M1. In addition, the absence of significant tACS effects indicates that whatever entrainment of neurons it may have elicited in the left M1, it did not manifest in meaningful effects on MEPs obtained from the right M1. This could either be because tACS had no effects on the right M1 at all or that any changes in oscillatory activity in right M1 did not also manifest in increased cortical excitability as measured by MEP amplitude.

In contrast, the current results align with a previous study from another research group that had a somewhat similar experimental design to the present one [24]. In this study, analogous TMS measurements were performed on the right M1 before and after anodal tDCS (1 mA) was applied to the left M1 using the SO-M1 montage. They found that left M1-tDCS did not significantly change MEP amplitudes evoked from the right M1 and recorded in the left hand immediately and 40 min after stimulation. Thus, the lack of change in MEPs recorded from the left hand after left M1-tDCS is consistent with the current findings. Together, these results suggest that left M1-tACS effects on cortical excitability are likely confined to the stimulated M1 both during and after stimulation. This interpretation would also align with the results of our previous M1-tDCS study that found no changes in MEPs of the right M1 during or after tDCS was applied to the left M1 for 20 min [25].

Indirect evidence from the literature is also consistent with the current findings. Although the absence of changes in MEPs from the right M1 was unexpected, it’s important to remember that 20–30% of M1-tDCS studies examining the excitability of the stimulated M1 have report no significant effects [6,7,8]. Therefore, it is not surprising that enhancing excitability in an unstimulated, distant area might be even more difficult, despite the strong anatomical connections between the hemispheres motor areas. Evidence of increased excitability in the right M1 would have supported the idea that tACS of the left M1 could enhance performance in both hands. For example, a motor learning study (without tACS) showed that practicing right-hand tasks improved left-hand skills [34]. This improvement was accompanied by increased right M1 excitability after several weeks of right-hand practice. Such findings suggest that the ipsilateral (right) M1 significantly contributes to voluntary movement control and motor learning for the right hand [13,31,32,33,34,35,36,37], even though it primarily controls the left hand. The current study indicates that tACS of the left M1 does not enhance right M1 excitability and may not influence motor learning in the ipsilateral right M1. However, this is speculative since no motor skill training was involved in this study.

### 4.2. Potential Explanations for the Absence of Left M1-tACS Effects on Right M1

tACS delivered to the left M1 using the typical SO-M1 montage could potentially modulate activity in the right M1 in several different respects based on simulated electrical field distributions [65] and known pathways between the involved brain regions: (1) the flow of current from the cathode placed over the supraorbital region to the anode placed over the left M1, which could then reach the right M1 through transcallosal connections; (2) the aforementioned current flow also produces an electrical field over the left premotor cortex (PMC), dorsolateral prefrontal cortex (DLPFC), and supplementary motor complex (SMC) in route from the cathode and anode. In turn, the activation of these areas could also ultimately modulate right M1 activity through their ipsilateral connections [66] to the left M1 (DLPFC, SMA, PMC) and the contralateral connections of left dorsal PMC to the right M1 [67,68,69]; and (3) the SO-M1 montage also generates an electrical field in the right hemisphere which is ipsilateral to the cathode. Although the electrical field is less over the right compared with the left hemisphere it could still modulate activity in the right DLPFC, SMA, PMC, and M1 (see Figure 1 in [65]). However, the exact physiological effects of the current flow in these particular circumstances and relative to right M1 have never been directly investigated.

In light of the three conceivable avenues through which left M1-tACS, administered at 1 mA utilizing the typical SO-M1 montage, could modulate the excitability of the right M1, the complete absence of changes in right M1 MEP amplitudes in this study strongly suggests that none of these pathways, nor their underlying mechanisms, significantly impacted right M1 function. Moreover, this lack of influence persists regardless of potential combinations or relative strengths of these pathways on right M1 excitability. Since this investigation did not have physiological measurements in addition to TMS to assess right M1 activity, exploring the reasons for the absence of left M1-tACS effects on right M1 fell beyond its intended scope. Nonetheless, drawing from existing literature, several potential factors contributing to these results can be cautiously considered.

One plausible explanation for the lack of significance observed in this study could be that tACS might not consistently induce increases in excitability to the extent observed in earlier studies. This idea gains support from an older M1-tDCS review, which noted a diminishing amount of tDCS effects on M1 excitability over time as study methodologies evolved [14]. Furthermore, a recent review, which specifically focused on M1-tDCS studies that failed to find significant effects on M1 excitability, cited almost 50 experiments that had studies indicating no substantial impact of M1-tDCS [6]. However, there have not been enough tACS studies conducted to date to be able to make the same kind of comparisons and conclusions as the tDCS related ones above. Another conceivable explanation for the absence of significant findings could be the presence of a significant proportion of tACS non-responders among the participants [60,70,71] as has been asserted in a series of tDCS studies on the topic. Nevertheless, Jonker et al. (2021) found no indication of the presence of either non-responders or responders in their extensive study [71]. These possibilities underscore the need for more research on these topics in tACS studies as there are limits to the inferences that can be drawn from tDCS studies due to the different underlying mechanisms of action by which the two stimulation techniques exert physiological and behavior effects in the motor system.

### 4.3. Study Limitations

The current study had a set of limitations that must be briefly addressed: (1) the study only investigated one possible set of tACS parameters of stimulation (e.g., electrode montage, frequency, current strength, stimulation duration, etc.). The current parameter set was chosen as they appeared to be the greatest effects on motor skill that were also supported by physiology recordings. Nonetheless, other sets of parameters used in other M1-tACS motor skill studies [7] such as tACS applied at either 10 or 20 Hz could have produced alternative findings. Thus, other sets of tACS parameter configurations that have shown effectiveness should be investigated in future motor skill and cortical excitability studies; (2) an experimental condition was not included that investigated the opposite arrangement of tACS delivered to the right M1 on left M1 activity. This could have produced different result due to the numerous intracortical and intercortical differences in organization between the two M1s; (3) the study did not include multiple TMS post-test blocks at various timepoints after M1-tACS administration ended to better characterize any longer-term after effects. For example, post-test of MEP amplitude could have been performed at intervals from five minutes up to 90 min after stimulation had ended [17]. However, it seems improbably that this testing would have yielded different findings relative than those obtained in the test block after tACS application, but this possibility cannot be completely ruled out and should be evaluated in future work; (4) MEP amplitude was the only physiological measurement taken during or after the stimulation. It would have been ideal to have performed paired-pulse TMS assessments of the intracortical neuronal populations that mediate measures such as short-interval intracortical inhibition (SICI), intracortical facilitation (ICF), or short-interval intracortical facilitation [72]; (5) a motor skill acquisition task was not incorporated, which could have been used to determine if the administration of tACS in these experimental conditions influenced the left hand motor performance and the functional relevance of any modulations in MEP amplitudes elicited from right M1; (6) despite the within-subjects design and the rather clear findings that tACS did not significantly enhance MEP amplitude compared with SHAM, the sample size could be viewed as relatively low. While small sample sizes represent a recognized limitation in neurophysiology and neuroscience research in general and across disciplines as outlined previously [73,74], the current sample was larger than the average tDCS study reported in review articles. For instance, see the tables in Buch et al. (2017) where the average sample size appears to be about 13 with many of the studies being between-subjects designs [12]. Nevertheless, the existence of many fewer tACS studies in the literature complicates such sample size comparisons and the sample size of the current study could therefore be viewed as an inherent limitation; (7) another limitation was that neuronavigation was not used in the study as our laboratory does not possess the equipment. Although some studies however have not showed an advantage to neuronavigation [75] compared to an experience TMS coil holder, other studies have indicated possible advantages. Therefore, as with the vast majority of TMS studies that have not used neuronavigation this will have to be viewed as an inherent limitation of the study; (8) tACS impacts on the stimulated left M1 were not undertaken in the study. This was the case for several reasons. The major obvious consideration was that the tACS electrode over the left M1 would preclude excitability measurements during stimulation as the TMS would have to be placed over the tACS electrode. In addition, if measurement of the stimulated hemisphere had been performed during the same experimental session it could have led to crossover (interhemispheric effects) that could have contaminated the contralateral measurements and also made the results ambiguous. Furthermore, MEP measurements much more difficult as it would have involved moving the coil back and forth to each side of the head in the same experimental session and could have led to more variable, error prone MEP measurements; and (9) a final limitation could be that interindividual variations in physiological, genetic, and anatomical factors on the susceptibility to tACS [45,46] could have played a role in the results. Although the within-subjects design allowed us to eliminate these factors in the individuals who participated in the study, it is nonetheless possible that the random sample had a large number of non-responders (if they even exist) to tACS. However, this issue has not been studied in nearly the detail as it has been in tDCS studies as discussed in the prior section above. This potential issue will have to be addressed in future tACS studies in both the stimulated and unstimulated hemispheres with large sample sizes similar the recent tDCS study by Jonker et al. (2021) [71].

### 4.4. Future Research Directions

There is substantial room for future research on the effects of tACS on cortical excitability and motor skill as far fewer studies have been performed on these topics relative to tDCS. In addition, there is almost an unlimited number of possible combinations of stimulation parameters and electrode montages that could be investigated. One issue that is receiving increasing attention in non-invasive brain stimulation studies is the potential differences in susceptibility to brain stimulation between individuals. Therefore, the parameters of tACS application may have to be customized depending on various anatomic and genetic characteristics of an individual. This will not only be challenging but also difficult to reconcile with the common competing viewpoint that non-invasive brain stimulation study protocols need to be more homogenous [15] to accurately establish the basic effects of a given tACS parameter space on the stimulated and unstimulated M1s and motor output of the two hands. Most importantly, these issues will be even more relevant for studies comprised of individuals with specific movement disorders characterized by a more affected hemisphere and associated hand such as focal hand dystonia, stroke, and Parkinson’s disease. An additional line of future inquiry would be the functional relevance of any MEP amplitude modulations on changes in motor skill. Although two early small-scale studies showed an association between increases in MEP amplitudes and increases in motor skill in stroke and Parkinson’s disease patients following M1-tDCS [76,77], more contemporary studies have reported a complete lack or a relationship [14,66,70,78,79]. Furthermore, similar studies have not been performed in response to M1-tACS. Moreover, it is more likely that changes in specific paired-pulse TMS measures such as SICI may be relevant to specific aspects of movement control. For example, SICI is involved in movement initiation [34], muscle force relaxation [80], and motor skill acquisition [81,82]. Once again there is much more research on the modulation of intracortical inhibitory and facilitatory circuits in response to M1-tDCS compared to tACS. Therefore, future studies could determine the influence of M1-tACS on these pathways in the context of motor skill acquisition in both the stimulated and unstimulated M1 and associated hand and arm system.

## 5. Conclusions

This study was a rationale initial exploration of possible modulations of right M1 excitability during and after application of left M1-tACS. Consequently, this investigation was conducted while the target muscle was at rest to isolate the effects of the stimulation, free from the influence of concurrent muscle activation or performance of a motor task. The novel findings produced by the study were that the MEP amplitudes evoked from the right M1 and acquired from the left hand did not differ significantly between the tACS and SHAM conditions at any of the specified points in time during or immediately after the stimulation was given to the left M1. Overall, the findings indicate that tACS applied to the left M1 does not significantly influence contralateral right M1 excitability during or immediately after stimulation, at least under the tACS parameters used in this study. Extensive future research is needed to gain a comprehensive understanding of tACS effects on both the stimulated M1 as well as the unstimulated contralateral M1. Such investigations should integrate diverse tACS paradigms with multiple physiological measurements and motor skill acquisition protocols involving the hands to elucidate the true potential of tACS as a method to enhance motor skill in different populations.

## Data Availability

The data presented in this study are available on request from the corresponding author. The data are not publicly available due to privacy and ethical restrictions.
